# Gender and education inequalities in dynapenia-free life expectancy: ELSI-Brazil

**DOI:** 10.11606/s1518-8787.2022056004025

**Published:** 2022-04-27

**Authors:** Viviane Santos Borges, Mirela Castro Santos Camargos, Fabíola Bof de Andrade

**Affiliations:** I Instituto René Rachou Fundação Oswaldo Cruz Belo Horizonte MG Brasil Instituto René Rachou. Fundação Oswaldo Cruz. Belo Horizonte, MG, Brasil; II Universidade Federal de Minas Gerais Escola de Enfermagem Departamento de Gestão em Saúde Belo Horizonte MG Brasil Universidade Federal de Minas Gerais. Escola de Enfermagem. Departamento de Gestão em Saúde. Belo Horizonte, MG, Brasil

**Keywords:** Muscle Strength, Life Expectancy, Aging, Hand Strength, Socioeconomic Factors, Cross Sectional Studies

## Abstract

**OBJECTIVE:**

To estimate the dynapenia-free life expectancy among community-dwelling older Brazilian adults and evaluate gender-related and educational differences.

**METHODS:**

This is a cross-sectional study. The data were obtained from the *Estudo Longitudinal da Saúde dos Idosos Brasileiros* (ELSI-Brazil – Brazilian Longitudinal Study of Aging), conducted from 2015 to 2016 in Brazil. Dynapenia is defined as low muscle strength (< 27kg for men and < 16kg for women), measured with a handgrip dynamometer. The dynapenia-free life expectancy was estimated using the Sullivan method based on the standard period life table and dynapenia prevalence, stratified by age groups, gender, and schooling.

**RESULTS:**

A total of 8,827 participants, aged 50 and over (53.3% women), were investigated. The prevalence of dynapenia was 17.7% among men and 18.5% among women. The women live longer and with more years free of dynapenia than men. Those in the higher education category (four or more years) presented an advantage in the dynapenia-free life expectancy estimates.

**CONCLUSIONS:**

The results of this study suggest the substantial impact of dynapenia on longer dynapenia-free life expectancy among older people. Understanding dynapenia prevalence and dynapenia-free life expectancy could assist in predicting care needs, as well as targeting efforts to delay the onset of complications related to it at older ages. Without the implementation of policy regarding dynapenia prevention, inequalities in health due to gender and socioeconomic status may continue to increase.

## INTRODUCTION

Worldwide, the populations are rapidly growing older^1,2^; the evidence, however, is inconsistent on whether additional years of life are accompanied by an increase of healthy ones^3^. Despite this being a worldwide achievement, aging is still an inexorable process that presents important economic and social challenges for all countries, such as the increase of chronic diseases and health-related long-term social care costs^6,7^, especially in developing countries where the majority of the world’s older populations live^8^. Aging aﬀects all organs and the musculoskeletal system is no exception. Older adults exhibit an increased risk of reduced muscle strength and power, a condition called dynapenia^9^.

Recent findings show a high prevalence of older adults living with dynapenia^12^. In high-income countries, such as England, the prevalence was found to be 12.3% among individuals aged 60 years^15^, whereas, in the United States, the prevalence among individuals 65 years or older was 44%^14^. In Brazil, almost one-fifth of older adults were classified as dynapenic, with reported rates for males and females estimated to be 16.6% and 17.7%, respectively^12^.

Dynapenia is associated with various adverse health outcomes including increased risk of falls^16,17^, functional^13,14^ and cognitive decline^18^, disability^14^, and mortality^15^. Furthermore, significant inequalities have been found concerning this condition, meaning that individuals in lower socioeconomic positions have greater negative consequences^12^.

Despite a large body of literature showing a strong link between dynapenia, socioeconomic status^12^, and mortality^15,19^, there is no evidence on how dynapenia affects the healthy life expectancy (i.e., indicator that combines disease and mortality information). An estimation of dynapenia-free life expectancy (DFLE), as well as other measures of disability-free life expectancy^7,20^, might provide information on the average number of healthy years lived among older adults, as well as give insights into the impact of health policies on the populations and develop a more informed health care response.

Given the high prevalence of this condition in the Brazilian population^12^ and the well-known inequalities within the country^21^, this study aimed to estimate the DFLE, by gender and education, among a representative national sample of community-dwelling older Brazilian adults.

## METHODS

This is a cross-sectional study with data obtained from the *Estudo Longitudinal da Saúde dos Idosos Brasileiros* (ELSI-Brazil – Brazilian Longitudinal Study of Aging), conducted from 2015 to 2016. This was a nationally representative population-based cohort study of non-institutionalized community-dwelling Brazilians aged 50 years and older, living in 70 municipalities, within the five major geographic regions of the country. The sample selection was based on a multistage area probability design, involving geographical stratification and clustering. All the details on sample design and methodology have been previously described^21^.

The ELSI-Brazil was approved by the ethics committee of the institution (protocol number: 34649814.3.0000.5091). All participants signed consent forms for the interviews and physical evaluation.

This study used data from 8,827 individuals aged 50 years or over, with full data on handgrip strength and other covariates.

Dynapenia was defined through the handgrip strength (i.e., maximal voluntary force)^9^, measured with a hydraulic handgrip dynamometer to assess isometric strength (SAEHAN Corporation, Korea, Model SH5001). Cut-off values for dynapenia were < 27 kg for men and < 16 kg for women^22,23^. In a sitting position, participants were instructed to squeeze the device with the dominant hand as hard as they could for two seconds. They were instructed to grasp the dynamometer in the hand and to keep their arms tight to the body with their dominant elbow forming a 90° angle. A familiarization test was first performed using the non-dominant hand^14^. The test was then performed three times with the dominant limb, with one-minute rest between tests, and the higher value of the three trials was used as the score.

### Statistical Analysis

We used the Sullivan method to compute DFLE and dynapenia life expectancy (DLE), as it can be applied with data from cross-sectional studies^24^. Dynapenia-free life expectancy and dynapenia life expectancy were estimated by combining the data from the life tables and current mortality experience among Brazilians of 2016, from the Brazilian Institute of Geography and Statistics (IBGE), with the prevalence of dynapenia among the population in the same period (ELSI-Brazil 2015–2016), thus estimating the number of years expected to be lived in a particular state of health. Dynapenia-free life expectancy was estimated by age, gender, and schooling. The following formula was used to estimate DFLE:


$$DFLE_{x}=\frac{\sum {\left({}_{n}\pi_{x}\right)}_{n}L_{x}}{I_{x}}$$

In which:

**DFLE**_**x**_ is dynapenia-free life expectancy, which comprises the average number of dynapenia-free years expected to be lived from age x.

_**n**_**π**_**x**_ is dynapenia-free prevalence in age group x to x+n.

_**n**_**L**_**x**_ is people-years lived from x to x+n, comprising the total number of years lived in each age interval.

**l**_**x**_**:** probability of living until age x.

Dynapenia life expectancy is obtained by subtracting dynapenia-free life expectancy from total life expectancy. In addition, we estimated the proportion of years expected to be lived with dynapenia based on the ratio between the number of years expected to be lived with it and the total number of years expected to be lived. Separate life tables were produced by age subgroups, gender, and schooling. The number of years lived in each age in the life tables was distributed according to point and interval estimates of the prevalence of dynapenia in each specific age group. All estimates generated were stratified by gender, age (in 5-year intervals), and education level (0–3 years and ≥ 4 years). We computed 95% confidence intervals (95%CI) considering the interval estimates of dynapenia prevalence.

The standard errors were calculated considering only the variance of the prevalence rates. To compare the DFLE between females and males and individuals with different schooling, we estimated their standard errors (or variances) and then the z-statistic to find the p-value. The test of the hypothesis of equality was based on the tables of Jagger 2006^25^. Data analysis was carried out using Stata version 12.1 (StataCorp, College Station, TX, USA) and Microsoft Excel 2010 (Microsoft, USA).

## RESULTS

Participants’ mean age was 62.5 years, 53.3% were female, and 32% had 0–3 years of education. Prevalence of dynapenia was of 18% among individuals aged 50 years or more, comprehending 17.7% among men and 18.5% among women. In individuals aged 50 to 59 years, the prevalence of dynapenia was of 10.9%, whereas in individuals over 80, the prevalence was of 55.6% (Table 1).

**Table 1:** Descriptive characteristics of participants by gender, age, schooling, and dynapenia. Brazilian Longitudinal Study of Ageing, 2015–2016 (n = 8,827).

Variable	%	95%CI
Gender		
Women	53.3	50.4–56.3
Men	46.7	43.7–49.6
Age (years)		
50–59	48.4	44.4–52.5
60–69	29.9	28.1–31.7
70–79	15.5	13.6–17.5
≥ 80	6.2	4.6–6.8
Schooling (years)		
0–3	32.4	29.1–35.9
≥ 4	67.6	64.1–70.9
Dynapenia total	18.1	16.3–20.0
Dynapenia men	17.7	15.6–19.9
Dynapenia women	18.5	16.3–20.9

95%CI: 95% confidence interval.

Figure shows the estimates for total life expectancy (TLE), DFLE, DLE, and proportion of years expected to be lived without dynapenia (DFLE[%]), for all age groups. The 50-year-old individuals could expect to live 30 years, of which 22.2 years would be lived without dynapenia, which accounted for 73.3% of TLE. In older adults with 80 years of age, these figures were 18.5 years for TLE, 11.3 years for DFLE, or 61.1% for DFLE[%].

**Figure:**
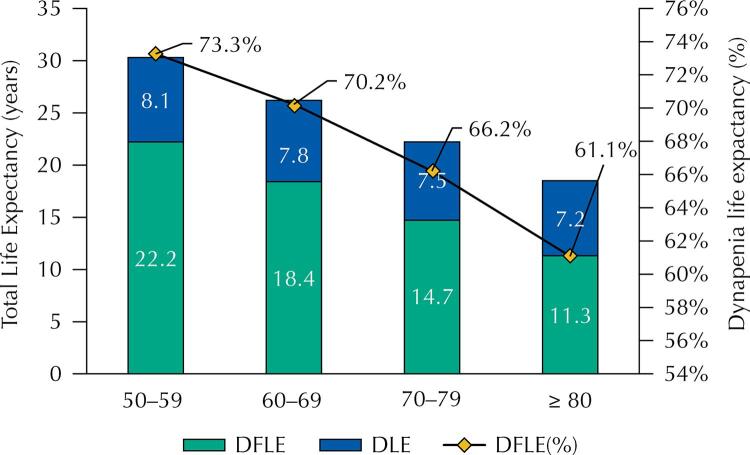
Estimates of total life expectancy (TLE), dynapenia-free life expectancy (DFLE), dynapenia life expectancy (DLE), and the proportion of years expected to be lived without dynapenia (DFLE[%]), by age, among older adults in Brazil. Brazilian Longitudinal Study of Ageing, 2015–2016 (n = 8,827).

Table 2 shows TLE, DFLE, and DFLE[%] by gender. TLE were lower for men than for women; for example, men in the age group of 60 and 70 years could expect to live an additional 20 and 13.6 years, respectively, of which 13.5 and 7.2 years will be lived without dynapenia, respectively. Among women of the same age groups, TLE at age 60 was 23.9 years and, at age 70, 16.3 years; the DFLE were 15.8 and 8.8 years, respectively (Table 2). Estimates of DFLE at the age of 50, 60, and 70 were significantly higher in women than in men. However, differences were not observed in the age group of 80 year.

**Table 2:** Estimates of dynapenia, total life expectancy (TLE), dynapenia-free life expectancy (DFLE), and the proportion of years expected to be lived without dynapenia (DFLE[%]), by age and gender, among older adults in Brazil. Brazilian Longitudinal Study of Ageing, 2015–2016 (n = 8,827).

Variable	Dynapenia (%)	TLE	DFLE	DFLE (%)	Dynapenia (%)	TLE	DFLE	DFLE (%)	DFLE comparisons p^a^
Age	Women				Men				
50–59	11.3	32.5	23.7	72.9	10.6	28	20.7	73.9	< 0.001
60–69	15.9	23.9	15.8	66.1	15.7	20.3	13.5	66.5	< 0.001
70–79	29.4	16.3	8.8	54.0	30.6	13.6	7.2	52.9	< 0.001
≥ 80	53.3	10.2	4.0	39.2	58.9	8.5	3	35.3	> 0.05

^a^ z-statistic.

The findings suggest inequalities in the prevalence of dynapenia for all ages, which was higher among older adults with lower educational levels. Educational differences in DFLE estimates were observed for all age groups (Table 3). Those with higher education levels (four or more years) had a higher DFLE than individuals with lower educational levels. For example, the DFLE is 5.9 percent points (1.3 years) higher for individuals aged 60 with four or more years of education than for those in the low education category, of the same age group.

**Table 3:** Estimates of dynapenia, dynapenia-free life expectancy (DFLE), and the proportion of years expected to be lived without dynapenia (DFLE[%]), by age and education, among older adults in Brazil. Brazilian Longitudinal Study of Ageing, 2015–2016 (n = 8,827).

Variable	Dynapenia (%)	DFLE	DFLE(%)	Dynapenia (%)	DFLE	DFLE (%)	DFLE comparisons p^a^
Age	0–3 years of education			4+ years of education			
50–59	14.6	21.4	70.6	9.9	23.0	75.9	< 0.001
60–69	19.6	14.1	63.5	13.7	15.4	69.4	< 0.001
70–79	33.0	7.7	51.0	27.0	8.7	57.6	< 0.001
≥ 80	57.0	3.2	34.0	53.9	4.2	44.7	< 0.001

^a^ z-statistic.

## DISCUSSION

This study used a large, nationally representative, sample of Brazilian older adults to estimate dynapenia and DFLE. We found that almost one-fifth of Brazilian older adults were classified as dynapenic, raising public health concerns due to its significant impact on disability, long-term care needs^14^, and mortality^15^. The findings suggest significant gender and educational inequalities in healthy life expectancy. The population in the higher education category (four or more years) had an advantage in the estimates of DFLE and women can expect to live more years without dynapenia than men.

During the last decade, a large body of literature evaluated trends and determinants for the unequal distribution of disability-free life expectancy among older adults in several countries, most of which used daily life activities as disability indicator^5,24,26,27^. To our knowledge, our study is the first to estimate the impact of dynapenia on healthy life expectancy. Although direct comparison between studies is difficult, the consistent association between functional performance and muscle strength^10,14^, and the role of the latter as a prognostic factor for disability and physical dependence among older adults^28^, indicate that the relationship between dynapenia and healthy life expectancy might share similar pathways, as it was suggested by previous disability measures.

Our study confirms the existence of gender inequality and corroborates previous studies which used disability as a health measure^7,24,26,27^. Although women of all ages live longer and have more years free of dynapenia, after the seventh decade men presented higher proportional dynapenia life expectancy than women. Accordingly, Ling et al.^10^ (2020) found that the decline in handgrip strength was steeper among men than women from age 85 to 89 years. Different to our findings, one study reported that women in Latin America and Asia spent more time in a state of disability and dependence than men, despite having longer life expectancies^7^. Freedman et al.^26^ (2016) also showed that the active years of older American women were no greater than the men’s, despite living longer lives. Similarly, Santosa et al.^27^ (2016) confirmed the existence of gender inequality in disability and life expectancy, in which they observed that women have longer life expectancy, but proportionally fewer years of disability-free life expectancy than men.

The differences across genders might be due to the biological and social determinants of healthy aging as women have higher prevalence of non-fatal chronic conditions compared with men, who are more likely to die from more fatal diseases before being disabled due to diseases^29^. Changes in healthy life expectancy can be driven by both changes in age-specific mortality and changes in disability prevalence. In women, for example, an increase in TLE combined with an increase disability prevalence may result in a greater number of years spent with disability. In contrast, a rise in mortality could reduce the number of years spent with disability, even with no changes to disability prevalence, thus reducing TLE while healthy life expectancy stays constant^3^.

Recent evidence suggests a strong link between socioeconomic status – particularly education – and disability-free life expectancy^3,30^. Our study corroborates previous findings^3,30,31,33^ suggesting that individuals with a higher level of education can expect to live a greater number of years without dynapenia. In Chile, data from a longitudinal study confirmed that the trajectories of health, disability, and mortality are more adverse for Chilean older people in the lower socioeconomic position^34^. In the United States, a recent study found an increase in education-based inequalities in TLE and a greater widening in education healthy life-expectancy, in which individuals with less than a high school education live, on average, less than those with a college education, and are also more burdened with disability^3^. Data from the Health and Retirement Study^30^ demonstrated that among older Americans with the same living arrangement, the higher educated live up to six years longer, in addition to eight more years in a disability free state and up to two fewer years in a disabled state. A previous study showed that educational attainment was responsible for a decline in the level of disability in the U.S. population from 1997 to 2010^33^.

Like previous studies, our findings underscore the important role of educational attainment in explaining trends in disability^30,32^ and healthy life expectancy^3,31^. Since causes of death moved from communicable to chronic diseases, researchers have argued that education is increasingly shaping one’s risk of disability and mortality^3^. Furthermore, higher levels of education are directly associated with the adoption of healthier lifestyles^5^, greater healthcare utilization^35^, and consequently effective management of chronic diseases^3,31,35^, which are closely related to disability^11^ and dynapenia^12^. Alternatively, the decrease in healthy life expectancy among less-educated individuals may be attributed to greater concentration of unhealthy behaviors^31^.

Among the strengths of this study, the use of a national representative sample to estimate dynapenia-free life expectancy might be acknowledged. Most of the previous studies, including the ones carried out in Brazil, evaluated limitations on basic and instrumental activities on daily living as the measure of disability. The use of dynapenia, which is closely related to disability, is particularly relevant for policy purposes, since it is directly linked to care needs, which is ultimately associated with economic burden^36^. A limitation of our study might be related to its cross-sectional design, despite using the Sullivan method, which is based on prevalence measures representing current health^37^. Additionally, this method is recommended due to its simplicity, relative accuracy, and ease of interpretation, which produces comparable results across many other countries.

## CONCLUSION

This study found significant gender and educational inequalities in the life expectancy of older adults. Women can expect to live more years without dynapenia than men. Regarding the socioeconomic status, the population in the higher education category had an advantage in the estimates of DFLE. These findings have implications for health policies directed towards predicting health care needs and preventing, or delaying, the onset of dynapenia. Further investigations are required to understand the underlying determinants and mechanisms of dynapenia on years of healthy life.
